# Zanubrutinib plus salvage chemotherapy for relapsed or refractory diffuse large B-cell lymphoma

**DOI:** 10.3389/fimmu.2022.1015081

**Published:** 2022-11-24

**Authors:** Xianggui Yuan, Xian Li, Yurong Huang, Xueli Jin, Hui Liu, Aiqi Zhao, Weiping Zhang, Wenbin Qian, Yun Liang

**Affiliations:** ^1^ Department of Hematology, the Second Affiliated Hospital, Zhejiang University School of Medicine, Hangzhou, China; ^2^ Department of Oncology, The Third Affiliated Hospital of Zhejiang Chinese Medical University, Hangzhou, China; ^3^ National Clinical Research Center for Hematologic Diseases, the First Affiliated Hospital of Soochow University, Hangzhou, China

**Keywords:** relapsed or refractory diffuse large B-cell lymphoma, zanubrutinib, Bruton’s tyrosine kinase inhibitor, combination chemotherapy, TP53, chimeric antigen receptor T-cell (CAR-T)

## Abstract

**Introduction:**

Relapsed or refractory diffuse large B-cell lymphoma (R/R DLBCL) has poor clinical outcomes when treated with conventional salvage chemotherapy. Monotherapy using zanubrutinib, a selective Bruton’s tyrosine kinase (BTK) inhibitor, has achieved modest antitumor effect in R/R DLBCL. Here we aimed to evaluate the efficacy and safety of zanubrutinib plus salvage chemotherapy in R/R DLBCL patients.

**Methods:**

We retrospectively reviewed R/R DLBCL patients who were administered with zanubrutinib plus salvage chemotherapy in our center between January, 2019 and December, 2021. Targeted panel sequencing of 11 lymphoma-related genes was performed on 8 patients with poor responses to zanubrutinib-based chemotherapy.

**Results:**

27 R/R DLBCL patients were enrolled. Median age at this study was 59 years (range, 15-72). The best overall response rate (ORR) was 74.1% and complete remission rate was 33.3%. With a median follow-up of 11 months (range, 1-17), the median progression-free survival (PFS) was 8.1 months, and the overall survival (OS) was not achieved. The most common grade-3/4 adverse events were neutropenia (70.4%), thrombocytopenia (66.7%), and febrile neutropenia (33.3%). In multivariate analysis, early treatment and overall response after chemotherapy were independent favorable prognostic factors for PFS. Overall response after chemotherapy was an independent favorable factor for OS. Among the 8 patients with poor response to zanubrutinib-based treatment, the majority of patients had *NOTCH2* mutations (n=8, 100%) and *TP53* mutations (n=7, 87.5%). However, these patients achieved an ORR of 75% at 3 months after CD19-CAR-T cell therapy (including 4 cases of complete remission and 2 cases of partial remission). With a median follow-up of 9 months from CAR-T cell infusion (range, 1-16 months), the median PFS was 14.5 months, and the median OS was not reached.

**Conclusion:**

With high efficacy and manageable tolerability, zanubrutinib plus salvage chemotherapy may be a potential treatment option for R/R DLBCL. CAR-T cell therapy may be a priority strategy for these poor responders to BTKi-based treatment.

## Introduction

Diffuse large B-cell lymphoma (DLBCL) is the most common aggressive lymphoma, accounting for 30% to 40% of non-Hodgkin lymphomas (NHLs) (1, 2). R-CHOP (rituximab, cyclophosphamide, doxorubicin, vincristine, and prednisone) has been the standard first-line treatment, achieving approximately 50%-60% of long-term remission. Unfortunately, up to 50% of patients are ultimately refractory to, or relapse after initial remission (3, 4). Salvage chemotherapy followed by autologous stem cell transplantation (auto-SCT) is effective for relapsed or refractory DLBCL (R/R DLBCL). But only 26% of patients respond to next-line salvage therapy and the median overall survival (OS) is only 6.3 months in the SCHOLAR-1 study (5). Currently there is no preferred salvage chemotherapy for R/R DLBCL.

Bruton tyrosine kinase (BTK) inhibitor has been proven highly effective for diverse B-cell malignancies. Ibrutinib, the first-in-class BTK inhibitor, has achieved an overall response rate (ORR) of 23% with modest activity in R/R DLBCL (6). Zanubrutinib (BGB-3111), a next-generation BTK inhibitor with minimal off-target effects, has demonstrated higher efficacy and safety for treating Waldenström macroglobulinemia, compared with ibrutinib (7). The phase 2 BGB-3111-207 study revealed that zanubrutinib monotherapy produced modest antitumor activity and favorable safety in R/R DLBCL, with an ORR of 29.3% and a complete remission (CR) rate of 17.1%. Developing mechanistically-based synergistic combinations may open a way to increase response rates and durability of zanubrutinib.

Over the last 2 years, our institution had integrated zanubrutinib into conventional salvage chemotherapy for R/R DLBCL. Therefore, we conducted a retrospective study to evaluate the efficacy and safety of zanubrutinib plus salvage chemotherapy for R/R DLBCL.

## Materials and methods

### Patients

This was a retrospective study of R/R DLBCL patients who received zanubrutinib plus conventional chemotherapy at our center. Patients were enrolled from January 2019 to December 2021. Clinicopathological data were collected using electronic medical records. Follow-up data was obtained from patients’ records or by telephone.

Patients were enrolled in this study who met the criteria as follows: age ≥14 years and histologically diagnosed CD20-positive DLBCL with relapsed or refractory disease. The excluded criteria were: central nervous system lymphoma, HIV-positive DLBCL, post-transplant lymphoproliferative disorders, or prior exposure to a BTK inhibitor. Refractory disease was defined as progressive disease (PD) or stable disease (SD) as the best response to chemotherapy or relapse ≤12 months after auto-SCT. Primary refractory DLBCL was defined as non-responders to first-line treatment or patients who relapsed within 3 months of CR or partial remission (PR). Relapse was defined as recurrence of progressive disease after achieving a CR through last-line therapy. Patients with incomplete medical data or those lost to follow-up were excluded from this study. The day of the last follow-up was January, 6th, 2022. The Ethics Committee of the Second Affiliated Hospital, Zhejiang University approved this study, which was conducted in accordance with the Declaration of Helsinki.

### Treatment

Salvage chemotherapy regimens included the ICE regimen (ifosfamide, carboplatin and etoposide), GDP regimen (gemcitabine, dexamethasone, and cisplatin) and GemOx regimen (oxaliplatin and gemcitabine). Salvage chemotherapy was selected by treating investigator and the same salvage chemotherapy had not been ever applied before they were enrolled in this study. Patients received rituximab when the patients relapsed >6 months after rituximab-containing treatment or based on their willingness. The dose was reduced by 20%-50% after patients experienced grade-4 adverse events (AEs). Prophylactic pegylated granulocyte colony-stimulating factor (Peg-G-CSF) was administered if grade-4 neutropenia or grade-3 neutropenia with fever developed in previous cycles of treatment. Zanubrutinib,160 mg orally, twice a day was initially given and the dose was reduced by 50% after patients experienced grade-4 neutropenia or grade-3 neutropenia with fever again in previous cycles of treatment after prophylactic Peg-G-CSF used. The number of cycles was up to 6 cycles with response. Autologous stem cell transplantation (auto-SCT) consolidation was recommended for transplant-eligible patients who achieved remission from combination therapy. Prophylactic antifungal therapy was not routinely used.

### Outcomes and toxicity assessments

Patients’ responses were assessed according to the revised response criteria for malignant lymphoma every two cycles (8). ^18^F-fluorodeoxyglucose positron-emission tomography and computed tomography (PET/CT) were used to assess responses after 4 cycles of treatment and upon suspected CR. Patients were regularly followed every 3 to 6 months thereafter. Overall response was defined as a PR or a CR. Covariates including disease stage, B symptoms, cell of origin, and results of immunohistochemical analysis were identified upon diagnosis. The nongerminal center B-cell like (non-GCB) or germinal center B-cell like (GCB) subtype was identified according to Hans’s algorithm. Eastern Cooperative Oncology Group (ECOG), Lactate dehydrogenase (LDH), extranodal sites, previous line of therapy, performance status, and disease status were determined before initiating treatment. Adverse events were evaluated according to the National Cancer Institute Common Terminology Criteria for Adverse Events version 5.0.

### Targeted panel sequencing

Targeted panel sequencing was performed using a selected panel that contained 11 genes related to DLBCL (NOTCH2, TP53, KMT2D, CD79B, TRAF3, PRDM1, MYD88, CD79A, CXCR4, ARIDIA and LYN). Genomic DNA was extracted from the formalin-fixed paraffin-embedded tumor tissue samples at recurrence or refractory disease. The detailed methods were carried out as described previously (9). The aimed average sequencing depth for all targeted regions was 2000×. Targeted panel sequencing and sequencing data analysis were performed by Idtbio Biotechnology Co. LTD (Hangzhou, China).

### Statistical analysis

Patients’ characteristics were summarized using descriptive statistical methods. Statistical analyses were performed using SPSS version 17. Statistical values were reported as medians. PFS was defined as initiation of zanubrutinib-based chemotherapy to disease progression or relapse, death of any cause, or last follow-up. OS was defined as initiation of zanubrutinib-based chemotherapy to death from any cause or last follow-up. PFS and OS were plotted according to the Kaplan-Meier method. Survival distributions were compared with the log-rank test. Multivariate analysis was performed using the Cox’s proportional hazards model. A two-sided P value <0.05 was considered a significant difference.

## Results

### Patients’ characteristics

We identified 27 patients who received zanubrutinib combined with salvage chemotherapies between January 2019 and December 2021. Patients’ baseline data are presented in **Table 1**. At the time of diagnosis, 74.1% (n=20) patients were presented with Ann-Arbor stages III-IV, 82.5% (n=23) were identified with the non-GCB subtype, 51.9% (n=14) patients exhibited double expression, and 14.8% (n=4) patients exhibited double-hit status. At the time of this study, the median age was 59 (range, 15-72 years), 55.6% (n=15) had an ECOG score of 2-4, 70.5% (n=19) had elevated LDH levels, 66.7% (n=18) had extra-nodal disease, 14.8% (n=4) had relapsed disease, and 85.2% (n=23) had refractory diseases, among which 48.1% (n=13) had primary refractory diseases. The median lines of prior chemotherapies were 2 (range, 1-4). Two patients received prior auto-SCT and one received prior CD19-targeted chimeric antigen receptor T-Cell (CAR-T) immunotherapy.

**Table 1:** Demographics and baseline characteristics.

Demographic or Characteristic		Cases (%), n=27	
Gender	Male	20	(74.1)
	Female	7	(25.9)
Age (years) at study entry	Median 59 (Range,15-72)		
	<60y	14	(51.9)
	≥60y	13	(48.1)
Hans classification	GCB	4	(14.8)
	Non-GCB	23	(85.2)
Double expression	Yes	14	(51.9)
	No	13	(48.1)
Double hit	Yes	4	(14.8)
	No	23	(85.2)
Ann arbor stage at diagnosis	I-II	7	(25.9)
	III-IV	20	(74.1)
B symptom at diagnosis	Yes	4	(14.8)
	No	23	(85.2)
ECOG at study entry	0-1	12	(44.4)
	2-4	15	(55.6)
LDH at study entry	Elevated	19	(70.5)
	Normal	8	(29.6)
Extra-nodal disease at study entry Bone		8	(29.6)
	Lung	6	(22.2)
	Liver	3	(11.1)
	Uterus	2	(7.4)
	Breast	2	(7.4)
Prior lines of therapy	1 line	7	(25.9)
	2 lines	12	(44.4)
	3 lines	6	(22.2)
	4 lines	2	(7.4)
Disease status	Refractory	23	(85.1)
	Primary refractory	13	(48.1)
	Relapsed	4	(14.8)
Prior auto-SCT		2	(7.4)
Prior CAR-T treatment		1	(3.7)

ECOG, Eastern Cooperative Oncology Group; LDH, Lactate dehydrogenase; auto-SCT, Autologous stem cell transplantation; CAR-T, Chimeric Antigen Receptor T-Cell; GCB, Germinal center B-cell like.

### Efficacy

**Figure 1:**
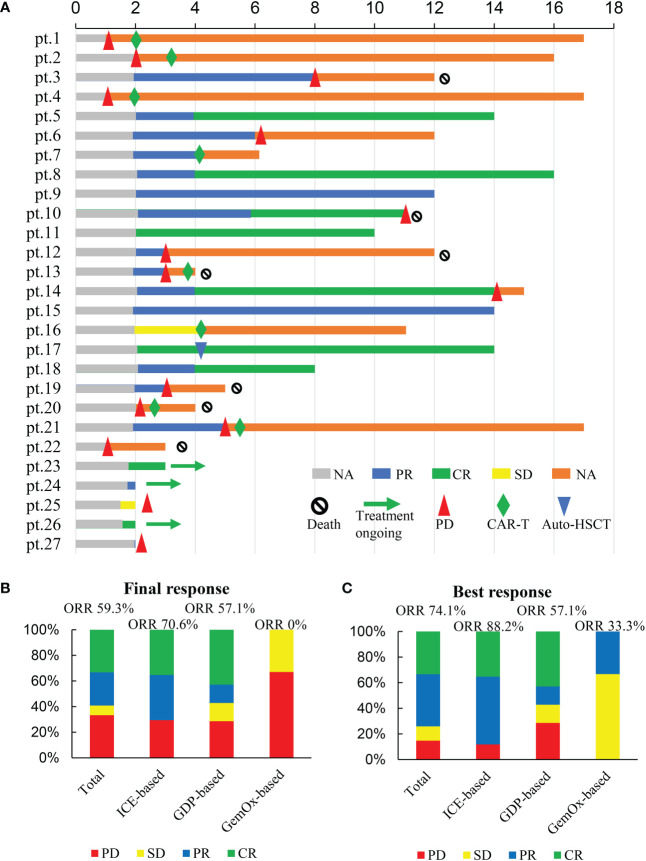
Responses to treatment. **(A)** swimmer plots of all patients evaluable for response; **(B)** Final responses to treatment; **(C)** Best responses to treatment.

Overall, 17, 7, and 3 patients received the ICE-based regimen, the GDP-based regimen, or the GemOx-based regimen, respectively. Swimmer plots of all patients evaluable for response are shown in **Figure 1A**. At the end of follow-up, 3 patients continued treatments, and 24 discontinued treatments. A total of 88 cycles of chemotherapy were administered. The median cycles of treatment were 4 (range, 1-6 cycles). 66.7% (n=18) patients received rituximab treatment.

**Figure 2:**
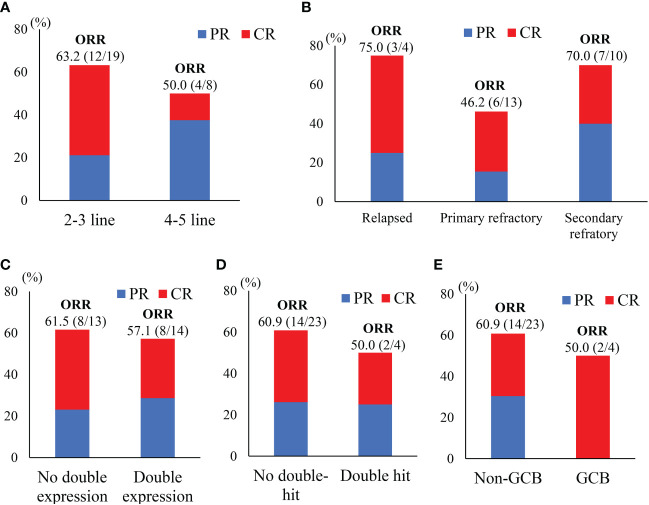
Subgroups responses to treatment. **(A)**Treatment lines; **(B)** disease status; **(C)** immunohistochemical analysis; **(D)** with or without double-hit; **(E)** cell of origin based on Hans’s algorithm.

The final responses and best responses to different combination regimens are shown in **Figures 1B**, **C**, respectively. At the end of treatment, 59.3% (n=16) patients had an overall response and 33.3% (n=9) achieved a CR. The best ORR was 74.1% (n=20) with 33.3% (n=9) of CR. Responses were observed in most subgroups (**Figure 2**), although there was a lower ORR trend in heavily pretreated patients (4-5 lines *vs.* 2-3 lines, 50.0% *vs.* 63.2%) and refractory patients (refractory *vs.* relapsed, 56.5% *vs.* 75%). Furthermore, 46.2% (6/13) patients with primary refractory DLBCL responded. The final ORR of the ICE-based, GDP-based, and GemOx-based groups were 70.6%, 57.1%, and 0%, respectively. The GemOx-based combination regimen was not as effective as the other two regimens. Overall, 7.1% (1/14) of transplant-eligible patients proceeded to auto-SCT, and 60% (6/10) of unresponsive patients as well as 2 PR patients proceeded to CD19-targeted CAR-T cell therapy with costimulatory 4-1BB endodomain (ClinicalTrials.gov ID: NCT04833504).

**Figure 3:**
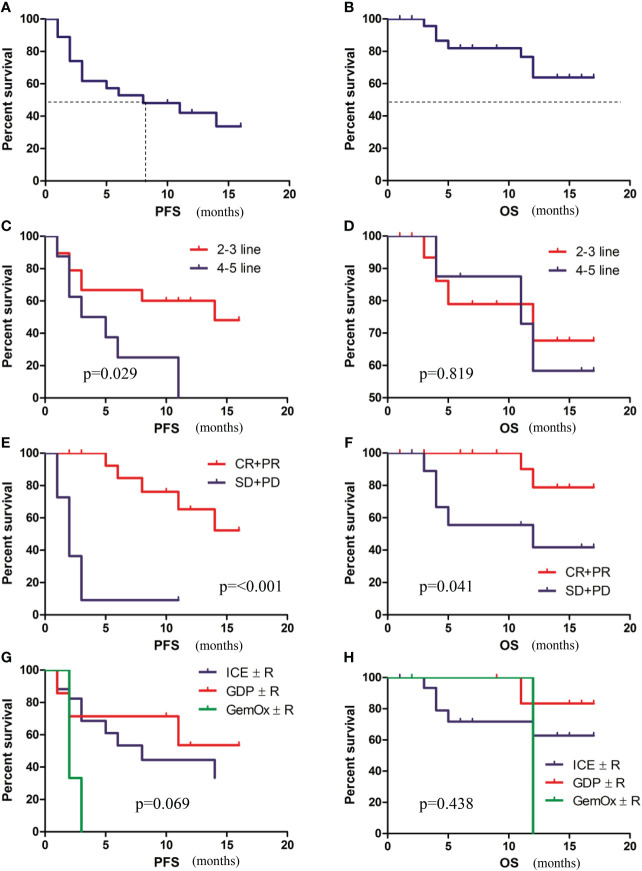
Survival analysis of patients. **(A)** PFS of patients. **(B)** OS of patients. **(C)** PFS of patients with early treatment (2-3 lines) versus late treatment (4-5 lines). **(D)** OS of patients with early treatment (2-3 lines) versus late treatment (4-5 lines). **(E)** PFS of patients with or without an overall response. **(F)** OS of patients with or without an overall response. **(G)** PFS according to combination regimens. **(H)** OS PFS according to combination regimens.

With a median follow-up of 11 months (range, 1-17 months), 15 patients progressed and 7 died. The median PFS was 8.1 months (95%CI, 0.2-15.8) (**Figure 3A**), but the median OS was not reached (**Figure 3B**). Univariate and multivariate analyses of PFS and OS are described in **Table 2**. PFS and OS did not differ significantly regarding cell of origin, age, serum LDH, ECOG, disease status, combination regimen (**Figures 3G, H**), and subsequent treatment. Univariate analysis revealed that PFS was significantly longer in patients with early treatment (2-3 lines vs 4-5 lines, p=0.029) (**Figure 3C**) and in those with overall response after chemotherapy (p<0.001) (**Figure 3E**). Furthermore, univariate analysis revealed that OS was significantly longer in patients with overall response after chemotherapy (p=0.041) (**Figure 3F**) but not with prior lines of chemotherapies(p=0.819) (**Figure 3D**). Multivariate analysis revealed that early treatment (HR=0.27, p=0.032) and overall response after chemotherapy (HR=0.06, p<0.001) were independent factors for favorable PFS. Overall response after chemotherapy (HR=0.11, p=0.036) was an independent indicator for favorable OS.

**Table 2:** Univariate and multivariate analyses of overall and progression-free survival.

	PFS						OS							
Variable	Univariate analysis			Multivariate analysis			Univariate analysis						Multivariate analysis		
	HR	95%CI	P-value	HR	95%CI	P-value		HR	95%CI	P-value		HR	95%CI	P-value	
Age ≥60y	0.75	0.24-2.13	0.573					1.19	0.26-5.33	0.819					
ECOG 2-4	1.45	0.51-4.09	0.468					5.39	0.65-44.9	0.075		5.17	0.50-53.1	0.162	
Elevated LDH	1.46	0.41-5.21	0.544					1.42	0.14-3.75	0.694					
Double expression	1.04	0.37-2.87	0.943					2.68	0.52-12.9	0.214					
Non-GCB cell of origin	0.42	0.13-1.35	0.927					0.49	0.09-2.54	0.604					
Combination Regimen	0.28	0.69-1.13	0.069	0.59	0.13-2.66	0.253		0.38	0.04-3.30	0.438		0.27	0.01-5.43	0.386	
Early treatment (2-3 line)	0.34	0.12-0.98	0.029	0.27	0.08-0.89	0.032		0.84	0.19-3.77	0.819		0.88	0.14-5.67	0.895	
Overall response at the end of treatment	0.11	0.03-0.38	<0.001	0.06	0.01-0.28	<0.001		0.22	0.04-1.12	0.041		0.11	0.02-0.87	0.036	
Subsequent CAR-T cell therapy or auto-SCT	0.96	0.34-2.70	0.633					0.36	0.07-1.84	0.323					

CI, confidential interval; HR, hazard ratio; ECOG, Eastern Cooperative Oncology Group; non-GCB, non-germinal center B-cell; LDH, lactate dehydrogenase; ORR, overall response rate; OS, overall survival; PFS, progression-free survival.

### Safety

Treatment-related adverse events are described in **Table 3**. Neutropenia (n=19, 70.4%) and thrombocytopenia (n=18, 66.7%) were the most common grade 3/4 adverse events. Febrile neutropenia was observed in 33.3% patients (n=9). Platelet transfusion was required for 7.4% (n=2) of patients. The most non-hematologic adverse events were fatigue (n=13, 48.1%), nausea and vomiting (n=14, 51.9%), and bleeding (n=5, 18.5%). One patient developed Grade 4 thrombocytopenia and gastrointestinal bleeding, which were resolved with active symptomatic treatments. Two patients developed grade 1 hematuria and the remaining 2 patients developed grade 1 petechiae when the platelet count was normal. These bleeding disappeared after symptomatic treatments. Atrial fibrillation, aspergillosis, and tumor lysis syndrome were not observed. All toxicities were manageable and reversible. No treatment-related deaths were observed. The categories and severities of adverse events did not significantly vary among the different combination regimens.

**Table 3:** Main adverse effects by treatment group.

**Toxicities**	**Total** **(n=27)**	**ICE-based** **(n=17)**	**GDP-based** **(n=7)**	**GemOx-based** **(n=3)**
**Hematologic, n (%)**				
Neutropenia	26 (96.3%)	17 (100%)	6 (85.7%)	3 (100%)
Grade 1-2	7 (25.9%)	3 (17.6%)	2 (28.6%)	2 (66.7%)
Grade 3-4	19 (70.4%)	14 (82.4%)	4 (57.1%)	1 (33.3%)
Anemia	24 (88.9%)	15 (88.2%)	6 (85.7%)	3 (100%)
Grade 1-2	16 (59.3%)	9 (52.9%)	4 (57.91%)	3 (100%)
Grade 3-4	8 (29.6%)	6 (35.3%)	2 (28.6%)	0 (0%)
Thrombopenia	24 (88.9%)	15 (88.2%)	7 (100%)	2 (66.7%)
Grade1-2	6 (22.2%)	5 (29.4%)	1 (14.3%)	0 (0%)
Grade3-4	18 (66.7%)	10 (58.8%)	6 (85.7%)	2 (66.7%)
Febrile neutropenia	9 (33.3%)	5 (29.4%)	4 (57.1%)	0 (0%)
**Non-hematologic, n (%)**				
Fatigue	13 (48.1%)	9 (52.9%)	3 (42.9%)	1 (33.3%)
Grade1-2	12 (44.4%)	8 (47.1%)	3 (42.9%)	1 (33.3%)
Grade3-4	1 (3.7%)	1 (5.8%)	0 (0%)	0 (0%)
Nausea and vomiting	14 (51.9%)	11 (64.7%)	2 (28.6%)	1 (33.3%)
Grade1-2	14 (51.9%)	11 (64.7%)	2 (28.6%)	1 (33.3%)
Grade3-4	0 (0%)	0 (0%)	0 (0%)	0 (0%)
Hepatotoxicity	1 (3.7%)	1 (14.3%)	0 (0%)	0 (0%)
Grade1-2	1 (3.7%)	1 (14.3%)	0 (0%)	0 (0%)
Grade3-4	0 (0%)	0 (0%)	0 (0%)	0 (0%)
Bleeding	5 (18.5%)	5 (71.4)	0 (0%)	0 (0%)
Grade1-2	4 (14.8%)	4 (57.1%)	0 (0%)	0 (0%)
Grade3-4	1 (3.7%)	1 (14.3%)	0 (0%)	0 (0%)
Atrial fibrillation	0 (0%)	0 (0%)	0 (0%)	0 (0%)
Diarrhea	0 (0%)	0 (0%)	0 (0%)	0 (0%)

### Targeted panel sequencing

**Figure 4:**
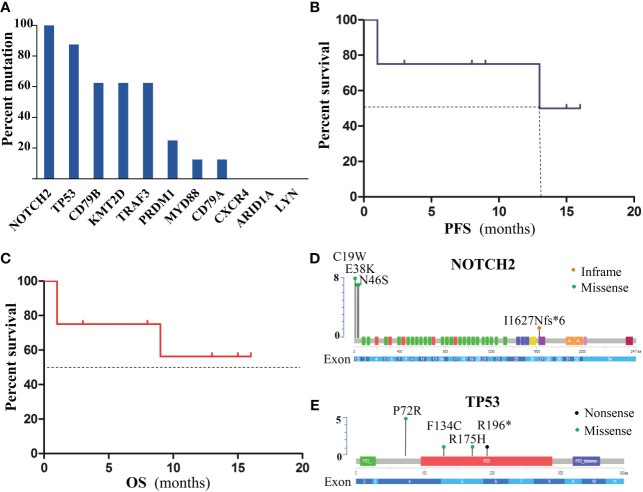
Gene mutations of patients with poor response to treatment. **(A)**Frequency of gene mutations. **(B)** PFS of patients. **(C)** OS of patients. **(D)** mutation location of NOTCH2 at the protein level. **(E)** TP53 Mutations at the protein level.

The gene panel was performed on 8 patients (6 PD and 2 PR with prior zanubrutinib- based chemotherapy), who proceeded to CD19-CAR-T cell therapy. Genomic DNA was extracted from the formalin-fixed paraffin-embedded tumor tissue samples at recurrence or refractory disease. In total, 52 somatic alterations were detected. The patients presented a median of 6 mutations per sample (range 2–9). Missense mutations were the most frequent at 50/52 (96.2%). **Figure 4A** present the gene mutation frequencies. The most frequently mutated gene was NOTCH2 (8/8) and TP53 (7/8). Mutation location of NOTCH2 and TP53 at the protein level are shown in **Figures 4D, E**, respectively. The next were mutations of KMT2D and CD79B, which observed simultaneously in 5 cases. MYD88 mutation was identified in only one case, who achieved partial response with zanubrutinib-based chemotherapy. The ORR at 3 months after CAR-T cell therapy were 75% (including 4 cases of CR and 2 cases of PR). With a median follow-up of 9 months from CAR-T cell infusion (range, 1-16 months), the median PFS was 14.5 months (**Figure 4B**), but the median OS was not reached (**Figure 4C**).

## Discussion

Zanubrutinib (BGB-3111) is a next-generation BTK inhibitor. Previous studies shows that zanubrutinib is more selective and active than ibrutinib in inhibiting BTK activity, with lower off-target activity against the follow protein tyrosine kinases: tyrosine kinase interleukin-2-inducible T-cell kinase (ITK), epidermal growth factor receptor (EGFR), and other kinases expressed in hepatocellular carcinoma (TEC) (10). In the present study, patients benefited from the encouraging efficacy of zanubrutinib plus salvage chemotherapy. The ORR of the present study was 74.1% and the CR rate was 33.3%. The median PFS was 8.1 months, but the median OS was not reached.

The responses and outcomes of BTK inhibitor monotherapy are unsatisfactory (6, 11). Developing mechanistically-based synergistic combinations may open a way to increase response rates and durability of BTK inhibitor. A study employing a high-throughput screening platform found that ibrutinib acted synergistically, additively, or both, with standard chemotherapeutic agents (12). A phase 1 study of ibrutinib plus R-ICE in R/R DLBCL demonstrated favorable tolerability and encouraging efficacies with 90% ORR and 55% CR (13). A phase 1/1b study of ibrutinib plus BR (rituximab, and bendamustine) induced 37% ORR and 31% CR in R/R DLBCL. Two patients with R/R DLBCL who received zanubrutinib plus R-DICE or R-DHAP respectively, also achieved a CR (14). Besides conventional salvage chemotherapy regimens, novel agents as BCL-2 inhibitors(venetoclax), immunomodulator(lenalidomide), PI3K inhibitors, XPO1 inhibitors(selinexor), IRAK4 inhibitors, immune checkpoint inhibitors, monoclonal/bispecific antibodies, CAR-T cell therapy and antibody-drug conjugates show strong synergistic activities with BTK inhibitors (12, 15, 16). A phase Ib study evaluated the combination of ibrutinib, lenalidomide, and rituximab for R/R DLBCL. The ORR was 44%, CR rate was 28%, and DOR was 15.9 months (17). Ibrutinib plus durvalumab achieved an ORR of 13% and 38% in GCB and non-GCB DLBCL, respectively (18). Ibrutinib plus venetoclax achieved an ORR of 53.8% after 4 cycles of treatment, and the median DOR、PFS and OS were 11 months, 5.6 months and 11.3 months, respectively (19). Acalabrutinib plus vistusertib (mTORC1/2 inhibitor) accomplished an ORR of 12% for R/R DLBCL (20). Ibrutinib plus buparlisib (a pan-PI3K inhibitor) for 37 patients with R/R DLBCL achieved an ORR of 31% (21). In the REAL-TREND study (22), a real-world retrospective analysis of treatment response of R/R DLBCL from 8 centers in China (including our center), the pooled ORR of salvage chemotherapy was 30% and the CRR was 9%. Our results with high efficacy indicated that zanubrutinib may act synergistically with conventional chemotherapeutic regimens.

DLBCL behaves genetic heterogeneity. Multiple studies have been made to identify sensitive patients who may potentially benefit from BTK inhibitors, based on tumor genetics, clinicopathology features, or both. Ibrutinib proves more effective in ABC-DLBCL (ORR=36.8%) than GCB (ORR=5%) (6). MCD, a genetic subtype of DLBCL with double mutant of *CD79B* and *MYD88*L265P, have inferior outcomes (23). The MCD subtype are more responsive to ibrutinib or zanubrutinib treatment (6, 11). *CD79B* and *MYD88*L265P double mutation are more responsive to ibrutinib, while single mutation is refractory (24, 25). Ibrutinib responders frequently harbor mutations in *KLHL14*, *RNF213*, and *LRP1B*, while non-responders commonly harbor mutations in *EBF1, ADAMTS20*, and *AKAP9* (26). Furthermore, mutation of *CARD11* and inactivation of TNFAIP3 (a negative regulator of NF-κB) predict no response to ibrutinib (6, 27). For non-responders to BTK inhibitors, *BTK* mutation are the best-described mechanisms (28). Third-generation non-covalent BTK inhibitors and CAR-T cell therapy, are promising strategies to overcome BTK inhibitor-resistance (29, 30). In the present study, the majority of the poor responders to zanubrutinib-based treatment had *NOTCH2* mutations and *TP53* mutations, while none was of MCD subtype, indicating that patients without MCD subtype maybe not benefit from BTKi-based treatment. *TP53* is a tumor suppressor gene and *TP53* mutation was an independent prognostic factor for survival in R/R DLBCL. In a retrospective study, in R/R DLBCL patients not treated with CAR-T cells, *TP53* mutation was an independent inferior prognostic factor for OS, but in the CAR-T cell group, this significance could not be shown (31). CAR19/22 T-cell therapy combined with ASCT is efficacious in r/r aggressive B-NHL with *TP53* alterations, producing a best ORR and CRR of 92.9% and 82.1%, respectively (32). However, in another retrospective study, *TP53* alterations (mutations and/or copy number alterations) were still associated with inferior CR and OS rates in R/R DLBCL treated with CD19-CAR-T treatment (33). In our study, among the 7 patients with *TP53* mutations, The ORR at 3 months after CAR-T cell therapy was 85.7% and the CRR was 57.1%. Compared with the results of TRANSCEND NHL 001study (Liso-cell) with the same costimulatory endodomain and similar follow-up time (ORR of 73% and CRR of 53%) (34), it seemed that the CAR-T cell therapy for patients with *TP53* mutations was still highly effective. Although the prognostic value of *TP53* mutations in R/R DLBCL patients receiving CAR-T cells is still undefined, CAR-T cell therapy may be a priority strategy for these patients.

As we expected, grade 3 and higher hematological toxicities were the major concern during and after zanubrutinib plus chemotherapy for patients with R/R DLBCL. Grade 3/4 neutropenia and thrombocytopenia of sole R-ICE regimen for R/R DLBCL occurred in 16% and 17.8% patients, respectively (35). Grade 3/4 neutropenia and thrombocytopenia of single zanubrutinib for R/R DLBCL occurred in 7.3% and 2.4% patients, respectively. In our present study, grade 3/4 neutropenia and thrombocytopenia of by zanubrutinib plus chemotherapy occurred in 70.4% and 66.7% of patients, respectively. Higher rates of hemorrhage were observed in 18.5% patients, which may be explained by thrombocytopenia and off-target activity of zanubrutinib. All above safety data showed that zanubrutinib plus chemotherapy increased myelosuppression. Fortunately, all hematological toxicities are manageable and reversible. There were no serious infectious complications or treatment-related mortality. To relieve bone marrow suppression, prophylactic Peg-G-CSF and recombinant human thrombopoietin (rhTPO) are required, and dose-modified salvage chemotherapy may also reduce hematologic toxicities. Regarding different BTK inhibitors, a phase 3 study demonstrated that the incidence and severity of BTK inhibitor toxicities were lower with zanubrutinib than ibrutinib in Waldenström macroglobulinemia (7). The efficacy and safety of zanubrutinib versus ibrutinib for CLL/SLL is ongoing in a head-to-head phase 3 study (36).

Our current study has several limitations. First, this study was insufficiently powered limited by the small sample size and short follow-up. Second, there were variations in prior therapies, which limit comparability. For example, only two patients received upfront auto-SCT in our study. Third, there were no genetic data available to demonstrate the underlying mechanisms of such synergistic combination. Nevertheless, the high activity of zanubrutinib combined with conventional chemotherapy, provides a new strategy for R/R DLBCL, and may serve as a bridge treatment to CAR-T cell therapy.

In conclusion, our study showed that zanubrutinib combined with salvage chemotherapy may serve as an effective salvage therapy for R/R DLBCL with manageable toxicity. Patients without MCD subtype maybe not benefit from BTKi-based treatment. CAR-T cell therapy may be a priority strategy for these poor responders to BTKi-based treatment. Further investigations of larger study populations are warranted to identify the most effective combination regimens and to precisely select patients.

## Data availability statement

The original contributions presented in the study are included in the article/supplementary material. Further inquiries can be directed to the corresponding authors.

## Ethics statement

The studies involving human participants were reviewed and approved by The Ethics Committee of the Second Affiliated Hospital, Zhejiang University. The patients/participants provided their written informed consent to participate in this study.

## Author contributions

Conceptualization, YL and WQ; Methodology, XY; Software, YH and XL; Validation, XL; Formal Analysis, YH and HL; Investigation, XY, XL, and YH; Resources, YL and WQ; Data Curation, HL and AZ; Writing – Original Draft Preparation, XY; Writing -Review and Editing, WZ, WQ and YL: Visualization, XJ; Supervision, YL and WQ; Project Administration, XY; Funding Acquisition, WQ. All authors contributed to the article and approved the submitted version.

## Funding

This work was supported by funds from Translational Research Grant of HCRCH (2020ZKZC01), the National Natural Science Foundation of China (No. 81830006), and the Natural Science Foundation of Zhejiang Province of China (No. LY15H160038).

## Conflict of interest

The authors declare that the research was conducted in the absence of any commercial or financial relationships that could be construed as a potential conflict of interest.

## Publisher’s note

All claims expressed in this article are solely those of the authors and do not necessarily represent those of their affiliated organizations, or those of the publisher, the editors and the reviewers. Any product that may be evaluated in this article, or claim that may be made by its manufacturer, is not guaranteed or endorsed by the publisher.
